# The Impact of Plant-Derived Polyphenols on Combating Efflux-Mediated Antibiotic Resistance

**DOI:** 10.3390/ijms26094030

**Published:** 2025-04-24

**Authors:** Anna Duda-Madej, Szymon Viscardi, Piotr Niezgódka, Wiktoria Szewczyk, Katarzyna Wińska

**Affiliations:** 1 Department of Microbiology, Faculty of Medicine, Wroclaw Medical University, Chałubińskiego 4, 50-368 Wrocław, Poland; 2 Faculty of Medicine, Wroclaw Medical University, Ludwika Pasteura 1, 50-367 Wrocław, Poland; szymon.viscardi@student.umw.edu.pl (S.V.); piotr.niezgodka@student.umw.edu.pl (P.N.); wiktoria.szewczyk@student.umw.edu.pl (W.S.); 3 Department of Food Chemistry and Biocatalysis, Faculty of Biotechnology and Food Science, Wrocław University of Environmental and Life Sciences, C. K. Norwida 25, 50-375 Wrocław, Poland; katarzyna.winska@upwr.edu.pl

**Keywords:** curcuminoid, efflux pump inhibitor, flavonoid, multidrug resistance, natural compounds, plant-derived, polyphenols, stilbenoid, tannin

## Abstract

The global healthcare system is increasingly challenged by the rising prevalence of multidrug-resistant bacteria and the limited therapeutic options for related infections. Efflux-mediated antibiotic resistance represents a significant obstacle, primarily due to the absence of drugs specifically designed to target bacterial efflux pumps. Recent research has identified polyphenols, a broad class of plant-derived organic compounds, as potential inhibitors of efflux pump activity. This review consolidates data on the inhibitory properties of eight widely distributed polyphenols: curcumin, quercetin, luteolin, tannic acid, naringenin, epigallocatechin-3-gallate, ellagic acid, and resveratrol. These compounds have demonstrated the capacity to inhibit efflux pumps, either through direct interference with bacterial protein function or by downregulating the expression of genes encoding pump subunits. Importantly, several polyphenols exhibit synergistic interactions with antibiotics, including colistin, ciprofloxacin, and tetracycline. For instance, quercetin has shown inhibitory potency comparable to that of established efflux pump inhibitors such as verapamil and reserpine. These findings suggest that polyphenols represent promising candidates for the development of novel efflux pump inhibitors. However, further research is required to validate their efficacy and safety and facilitate their translation into clinical applications for combating antibiotic resistance.

## 1. Introduction

Efflux pumps are protein channels located in the cell membrane. They are responsible for removing dangerous substances from the cell, i.e., antibiotics, heavy metals, detergents, toxins, and some active agents of disinfectants and antiseptics. Due to their different specificities and substrate ranges, they exhibit two types of mechanisms of action: specific (removal of defined substrates) and non-specific (removal of several different compounds) [1]. Pumping systems are most often encoded on chromosomes (expression occurs by substrate induction or mutation associated with environmental stress) [2]. However, genes encoding transport proteins can also be found on plasmids and/or other mobile genetic elements, i.e., transposons and integrons [3,4]. This is particularly dangerous as these elements also carry antibiotic resistance genes, which promote the formation of multidrug-resistant (MDR) strains [5].

**Figure 1:**
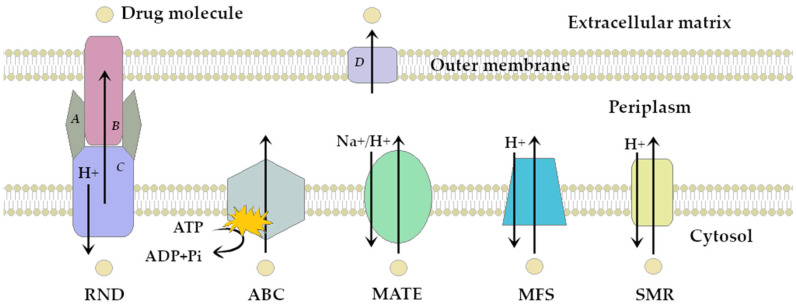
Simplified scheme of efflux pumps classification and mechanism of action; *A*—membrane fusion protein, *B*—outer membrane protein, *C*—H^+^ gradient-dependent efflux pump protein, *D*—outer membrane protein.

The pumping out mechanism depends on the structure of the bacterial membrane and cell wall. Transport proteins are classified into five families according to the number of transmembrane sequences, substrate specificity, mechanism of action, and function, as follows: (i) MFS—major facilitator superfamily [6,7]; (ii) SMR—small multidrug resistance [8,9]; (iii) ABC—ATP-binding cassette; (iv) MATE—multidrug and toxic compounds extrusion, and (v) RND—resistance-nodulation-cell division [10,11,12]. Figure 1 shows a simplified scheme of action for all five efflux pump families.

In Gram-negative bacteria, owing to the more complex structure of their cell envelope, efflux transporters form a triple structure composed of: (i) an intracellular cytoplasmic membrane protein; (ii) an outer membrane protein forming a transport channel, and (iii) a periplasmic space protein connecting (i) and (ii) [13,14,15]. For these bacteria, the RND and MATE families are more significant. In contrast, Gram-positive bacteria, characterized by a less complex structure, have a transport system represented by a single membrane protein [16]. All five families can be found in these bacteria; however, the most common are MFS, SMR, and ABC. Table 1 summarizes the characteristics of efflux pump families among Gram-negative and Gram-positive bacteria.

The phenomenon of natural pumping out for antibiotics not only gives bacteria resistance properties, but can also lead to the development of new resistance mechanisms by (i) changing the target site of action of antimicrobial drugs, and (ii) producing degrading enzymes [43,44,45]. Consequently, the expression of efflux mechanisms can lead to the interaction of different resistance mechanisms and initiate the emergence of multidrug-resistant bacterial strains, posing a problem for modern medicine. The fact that the efflux pump mechanism can be modified extremely easily and quickly becomes dangerous. Indeed, a single mutation in the genome of a bacterial cell contributing to the overexpression of new, unknown pumps is enough [46,47]. The rapidity of this adaptation is becoming the bacteria’s weapon of choice against commonly used antibiotics.

**Figure 2:**
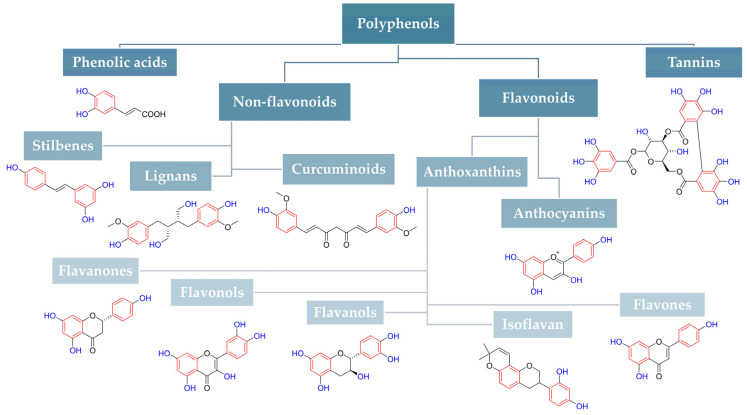
Classification of natural polyphenolic compounds based on structure.

The discovery of effective methods to inhibit efflux pumps is becoming a challenge for many scientific fields. This is an extremely difficult task due to the fact that several independent mechanisms may be involved in pumping out a single antibiotic. The known efflux pump inhibitors include: (i) reserpine [48]; (ii) berberine (BRB) [49,50]; (iii) 5’-methoxyhydnocarpin (5’-MHC) [51]; (iv) quinoline derivatives [52]; and (v) arylpiperidine and arylpiperazine [53,54]. However, most of them are not neutral to the human body and are characterized by side effects, including high toxicity. Therefore, the identification of new efflux pump inhibitors (EPIs) is necessary. Great hope is placed in natural compounds, specifically their main components, active substances. Given their diverse and highly selected activity, they can inhibit modification of the efflux pump mechanism formed by random mutations in the bacterial genome. One of the promising groups capable of showing high specificity in recognizing efflux pump substrates is polyphenols. These compounds are widely distributed in nature in many forms (Figure 2). They are found in green tea, Yerba mate, cocoa, olive oil, nuts, legumes, fruits, as well as wine and beer [55,56]. Their chemical structure, consisting of a hydroxyl group linked to an aromatic ring, influences their importance in the body’s purification and regeneration processes through strong antioxidant and free radical-destroying activities [57]. In addition, the most important positive effects of polyphenols on the human organism include: (i) antioxidant activity [58]; (ii) impact on the cardiovascular system, [59,60,61]; and (iii) effects on the immune system [62,63], including (1) anti-tumor [64,65], (2) antiviral [66,67], (3) antibacterial [68,69,70], (4) antifungal [71,72], and (5) analgesic [73,74] effects. Considering their wide distribution and wide-ranging effects, polyphenols are exactly the group of compounds that should be seen as potential inhibitors of efflux pumps or as compounds capable of avoiding membrane transporters.

Our study focused on reviewing the ability of polyphenols to overcome efflux-mediated antibiotic resistance among human pathogenic bacteria. For clearer communication of this very broad topic, we have divided our results into sections based on the main classification of polyphenols shown in Figure 2. In turn, each section clusters subsections describing specific types of microorganisms. In addition, due to the growing recent interest in all kinds of carriers with applications in phytomedicine, we have separated the results focused on the anti-efflux properties of polyphenols’ derivatives and nanoparticles (NPs). All the studies we cite were carried out only in vitro/in silico.

The main aim of our review is to highlight the importance of the efflux mechanism in the context of increasing antibiotic resistance worldwide. Efflux is one of the main mechanisms responsible for multidrug resistance in both Gram-negative and Gram-positive bacteria. Efflux pumps are not specific to a single antibiotic, but can simultaneously remove different groups of antibiotics from the cell using different action mechanisms, contributing early on to the failure of the applied therapy. It is important, as we underline in this review, that the effect of efflux pumps is not total insensitivity to a given microorganism, but low-level resistance. This phenomenon promotes the selection of increasingly resistant strains, and its detection is overlooked in routine diagnostics. Surprisingly, pathogens gain the ability to efflux various antibiotic molecules from their cells through the expression of the pumps mentioned above. So far, a reflection of the scale of this problem has been seen in the global increase in colistin (COL) resistance by efflux mechanism, among other things. The widespread use of linezolid to treat infections caused by Gram-positive strains, i.e., MRSA (methicillin-resistant *S. aureus*) or VRE/GRE (vancomycin/glycopeptide-resistant *Enterococcus*), has also contributed to the emergence of the latest resistance phenotype LR (linezolid resistant). It is associated with the presence of *optr*A and *poxt*A genes, encoding the ABC family of transporter proteins in *Enterococcus* spp. that condition the LRE phenotype (linezolid-resistant *Enterococcus*) [75,76]. Mechanisms involving MFS and ABC family pumps have also been identified in *S. aureus* and *Staphylococcus epidermidis* [77]. This review is intended to increase the understanding of the role of efflux in resistance and, consequently, enable the selection of antibiotics that are not substrates of efflux pumps or combination therapies with EPIs.

Our idea was to develop a review that comprehensively summarizes the available reports on the activity of a range of polyphenol groups in terms of their properties as inhibitors of efflux pumps in bacteria. We tried to include the greatest possible number of subfamilies of substances contained in this broad group, resulting in a thorough yet complex study. We also aimed to highlight pioneering pharmaceutical solutions—the development of derivatives of these substances and nanoparticles. As a result, to the best of our knowledge, we have created the first review article that not only attempts to describe the activity of pure substances of natural origin but also underlines the extraordinary potential of these compounds as precursors of a new generation of antimicrobial pharmaceuticals.

## 2. Anti-Efflux Potentials of Pure Polyphenols

### 2.1. Curcuminoids Activity as an Efflux Pump Inhibitor

**Figure 3:**
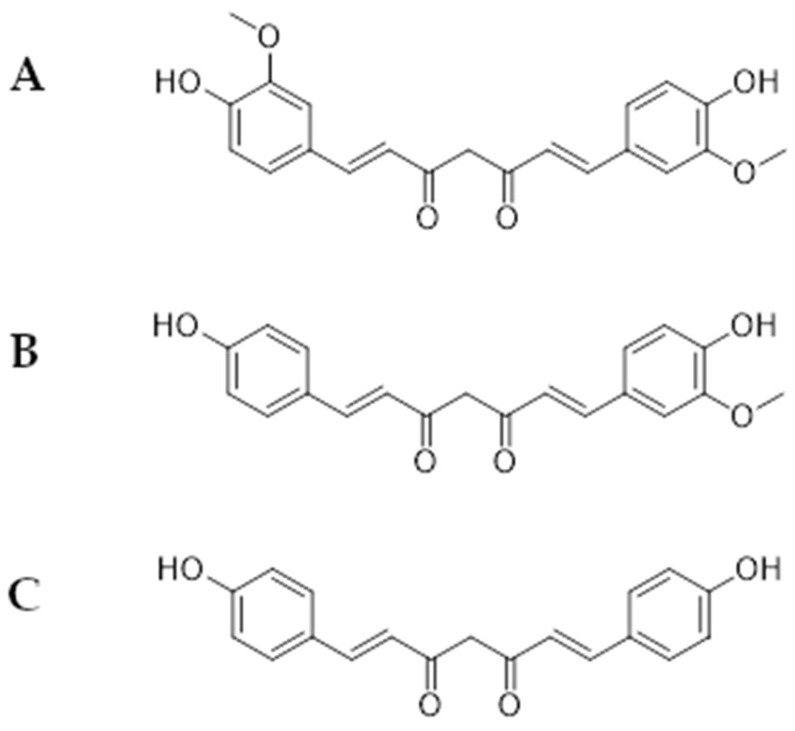
Molecular structure of curcuminoids (two-dimensional); (**A**)—curcumin, (**B**)—demethoxycurcumin, (**C**)—bisdemethoxycurcumin.

Curcuminoids (Figure 3) are a group of polyphenol compounds originally derived from rhizomes of *Curcuma longa* L. as well as other members of the *Zingiberaceae* family [78]. These plant-derived compounds form a group of substances (diarylheptanoids, derivatives of hydroxycinnamic acid) consisting of three substances: curcumin (A), demethoxycurcumin (B), and bisdemethoxycurcumin (C) [79].

Curcumin (CU)-[1,7-bis (hydroxyl-3-methoxyphenyl)-1,6-heptadiene-3,5-dione] is a polyphenol compound that has been identified as a possible EPI molecule. Additionally, the substance is widely known for its anti-inflammatory [80], anti-bacterial [81], anticancer [82], and neuroprotective [83] effects.

**Non-fermenting rods:** Promising reports on CU activity as an EPI come from the Negi et al. study [84]. The researchers reported that the exposure of MDR *Pseudomonas aeruginosa* strains to polyphenols resulted in a reduction in the percentage of bacterial strains resistant to classic antibiotics used to treat infections with *P. aeruginosa* etiology. Furthermore, the strains were tested, and it was shown that 9–26% of them were not susceptible to the tested antibiotics (meropenem, ceftazidime, carbenicillin, gentamicin, and ciprofloxacin) and were characterized by resistance due to the efflux mechanism (phenylalanine-arginine β-naphthylamide [PAβN] assay). CU in the concentration range of 20–50 μg/mL led to a restoration of ceftazidime (CAZ) sensitivity at a comparable rate to that of PAβN. In addition, the efficiency of CU in this process was superior to comparators in the cases of carbenicillin, gentamicin (GEN), and ciprofloxacin (CYP) [84]. CU was also tested for its ability to restore aminoglycoside sensitivity in *P. aeruginosa* strains that were originally resistant to them. For 100 of them, MICs for amikacin (ACN), tobramycin (TOB), and GEN were tested before and after the addition of efflux pump inhibitors (CU or PAβN). Importantly, it was shown that an above threefold reduction in MIC for any antibiotic was more frequent with polyphenol than with the comparator: GEN—26.6% vs. 6.6%, TOB—16.6% vs. 5.5%, and CAN—27.2% vs. 9.09% of tested strains. Surprisingly, the study proved that CU is a much more active inhibitor of efflux pumps than PAβN and can successfully restore aminoglycoside sensitivity in originally resistant *Pseudomonas* spp. [85]. The ability of CU to overcome efflux-mediated antibiotic resistance in *P. aeruginosa* was investigated by Sulaiman et al. [86]. Their study showed a high percentage of resistance to CAZ (66%) and CYP (40%) among the bacteria; a total of 42% of strains were defined as MDR (total number equal to 74). The susceptibility of 13 MDR strains to CYP was significantly increased by exposure to CU at a concentration of 50 µg/mL (decrease in MIC from 1- to 5-fold). However, a similar phenomenon was not observed when CU and CAZ were combined. The study confirmed the previously cited reports on the ability to disrupt the outflow of fluoroquinolones in pathogens previously resistant to them; however, clinical usage of CU as an EPI requires further research [86]. Another study on the ability of CU to disrupt efflux in *P. aeruginosa* showed that the exposure to polyphenol increased the diameter of the growth inhibition zone in the Kirby–Bauer test. What is particularly important is that 100% of the strains tested showed expression of the pump-associated *mex*B gene. The strains resistant to meropenem (MER), CYP, ACN, and CAZ treated with CU have acquired sensitivity to aminoglycoside (increase of ZOI from 0 mm to 10 mm at exposures of 25–50 μg/mL of CU) and fluoroquinolone (ZOI increased from 0 to 7 mm in 50 μg/mL of CU). The presence of the efflux pump and the documented role of CU in its dysfunction adequately explain the increasing sensitivity of *P. aeruginosa* to the antibiotics used in combination [87]. The effect of *A. baumannii* exposure to CU alone and CU in combination with COL was assessed as a strategy to break down the reduced susceptibility of the pathogen to the antibiotic due to the efflux mechanism. A synergistic interaction of CU + COL was demonstrated in the checkerboard test. Polyphenol at 100 µM lowered the MIC for the antibiotic threefold, from 2 to 0.5 μg/mL. In turn, in the analysis using ethidium bromide (EtBr)—a well-established method to monitor efflux activities in bacteria—the use of CU led to the accumulation of the compound in a manner similar to the exposure to carbonyl cyanide m-chlorophenyl hydrazone (CCCP). In addition to increased accumulation, a decrease in EtBr outflow after exposure to CU was noted, and at the molecular level, a decrease in *emr*B gene expression (gene encoding a compound of an efflux pump) was demonstrated. The reduction in expression was enhanced with the use of a synergistic combination. Importantly, CU also inhibited efflux ability via transporters other than AdeABC, because the tested *A. baumannii ΔadeB* mutant also presented the accumulation of EtBr and a decrease in EtBr outflow [88].

***Enterobacteriaceae***: The effect of CU on efflux pumps produced by representatives of the *Enterobacteriaceae* has also been demonstrated. One study showed that exposure of COL-resistant *E. coli* strain U3790 and *K. pneumoniae* BC936 to CU, among others, enabled increased EtBr accumulation inside cells. Of all the organic compounds tested, CU was inferior only to CCCP in potentiating efflux inhibition. What is more, a checkerboard analysis was carried out between COL, CU, and an inhibitor of MarR operon (operon associated with the overexpression of the AcrAB-TolC efflux pump). The test also used knock-out mutants of *acr*A, *acr*B, and *tol*C genes. The study showed a strong synergy of CU with both preparations (32-128-fold reduction in MIC value for COL). The ability to also lower the MIC against *acr*B mutants indicated that CU may interfere with the efflux via other pump functions, e.g., EmrAB-TolC [89]. Qin et al. also investigated interactions between CU and COL and the potential clinical implications of polyphenols’ ability to restore COL susceptibility in MDR Gram-negative bacteria [90]. Synthesized liposomes containing a CU + COL combination were characterized by such high activity against COL-NS (non-susceptible) *E. coli* (decrease in MIC for the antibiotic ≥31 times) that the interaction was considered synergistic (checkerboard assay). The analysis using EtBr and CCCP as a comparator showed that CU in liposomal form led to the accumulation of EtBr inside *E. coli* cells in a more efficient way than CCCP (fluorescence analysis). The results of the study clearly indicated that CU interfered with MDR pumps in *E. coli* and thus intensified the effect of classic antibiotics [90]. Liang et al. evaluated the effect of curcumin photosensitization on survival and gene expression in *Shigella flexneri*. Interestingly, exposure to light and CU led to the upregulation of key genes regulating the functions of the AcrAB pump: *mar*A and *arc*A. This may suggest that while CU (as other studies have shown) is able to inhibit efflux pumps in a number of pathogens, it can also induce its overexpression, especially under oxidative stress conditions [91].

***Staphylococcus aureus***: Joshi et al. examined the activity of CU against four *S. aureus* strains characterized by efflux resistance conditioned by pumps: NorA (resistance to CYP), TetK (resistance to tetracycline [TET]), MsrA (resistance to erythromycin), and MdeA (resistance to mupirocin). Exposure to CU led to an 8-fold reduction in MIC for CYP, which was associated with overcoming resistance conditioned by the NorA pump. However, the compound was unable to interfere with the remaining pumps [92]. The ability of CU to inhibit the activity of the *S. aureus* NorA pump and thus restore sensitivity to fluoroquinolone preparations was the main thread of the Jaberi et al. study [93]. The researchers showed that while 31% of tested strains (out of 100) were resistant to CYP, the addition of CCCP significantly reduced the MIC for the antibiotic in 13 cases, proving resistance in the efflux mechanism. After using CU, interesting molecular effects were noted—the level of *nor*A gene expression decreased in the range of 3–100 times, depending on the strain. Similar to CCCP’s impact on CYP activity, a decrease in MIC for the antibiotic was also noted [93].

### 2.2. Flavonoids Activity as an Efflux Pump Inhibitor

**Figure 4:**
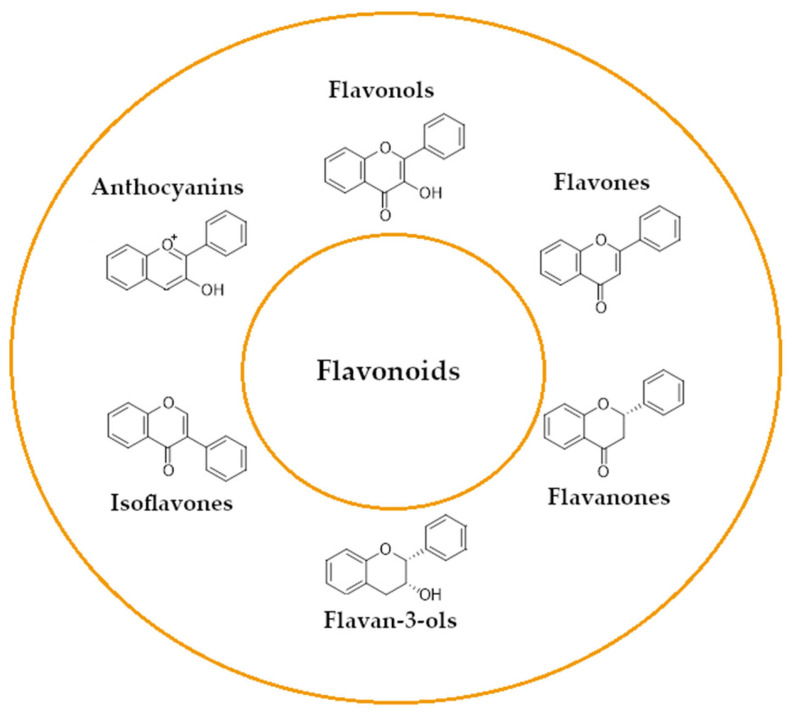
Flavonoids classification with regard to their molecular structure (two-dimensional).

A wide range of plant-derived polyphenolic compounds characterized by various biological effects were identified to be flavonoids. Flavonoids present as a large group of plant secondary metabolites with a common carbon-based skeleton composed of three rings (C6-C3-C6) and modified via alkyl and hydroxide groups [94]. The main division of flavonoids into subgroups is presented in Figure 4.

After reviewing the literature on the EPI properties of flavonoid substances, we focused our attention on four compounds with promising interference profiles with bacterial efflux. The detailed descriptions of flavonoids occurrence and documented biological activity are summarized in Table 2. In the following part of this section, we present the collected data associated with their EPI properties.

#### 2.2.1. Quercetin Activity as an Efflux Pump Inhibitor

**Gram-positive bacteria:** Quercetin’s (QR) activity of NorA efflux pump inhibition in *S. aureus* was assessed in the Brown et al. study. The researchers showed that the preparation was not characterized by a significant inhibitory effect of efflux, reaching IC_50_ = 75 µg/mL. A similar result was recorded against other polyphenols, including luteolin. The comparator used in the study—CCCP—was the most active inhibitor (IC_50_ = 4.7 μg/mL) [123]. QR and a number of other flavonoids were tested in the field for their ability to intensify the action of classic antibiotics against (ampicillin, CYP, erythromycin, oxacillin, and TET) bacteria originally resistant to them via the efflux mechanism. The study tested clinical *S. aureus* and selected mutants overexpressing *nor*A, *tet*K, *msr*A genes. In the study, the QR (500 mg/L) and its isomer, morin, were found to sensitize CYP-NS *Staphylococcus* strains to the antibiotic (strain SA1199B overexpressing pump genes). In addition, the ability of QR and morin to reduce MICs for CYP, TET, and erythromycin (3-16-fold reduction) was proven. The described interaction was confirmed in relation to pathogens forming efflux pumps. Importantly, the study indicated the existence of the mechanism of polyphenols’ interference with the mentioned pumps [124]. Abreu et al. continued their research on the ability of QR and morin to interfere with biofilm formation by *S. aureus* SA1199B strain (overexpression of NorA pump). According to the previous study [125], QR efficiently increased CYP activity in a strain originally resistant to the mentioned drug. Morin, on the other hand, was characterized by the ability to inhibit the development of biofilm not only in the SA1199B strain, but also in two other strains characterized by overexpression of the TetK pump that is associated with resistance to TET and the erythromycin-resistant strain (MsrA) [125]. The ability of QR to overcome antibiotic resistance in *S. aureus* by interfering with efflux pumps NorA, MrsA, and TetK was also examined. There was a spectacular reduction in MIC for TET (from 1024 to 128 μg/mL) when the mentioned drug was applied together with QR, and a statistically significant reduction in MIC for CYP (from 256 to 128 μg/mL). Additionally, the EtBr assay confirmed a reduction in MIC for the substance in relation to the tested strains (strains with TetK and NorA overexpression), which was associated with the inhibition of efflux pumps. A ligand/protein complex stabilized by hydrogen bonds formed in silico showed QR’s ability to directly bind in the NorA efflux pump area [126].

**Gram-negative bacteria**: Pal et al. demonstrated the efficacy of QR as a formulation capable of inhibiting the activity of both carbapenemases in carbapenem-resistant Gram-negative bacilli (CR-GNB) and overcoming the efflux pump resistance. Importantly, the tested polyphenol showed a synergistic interaction with MER. Analysis using EtBr evaluated the effect of QR on *E. coli* and *K. pneumoniae* overexpressing the acrB gene, *P. aeruginosa* (gene *mex*B), and *A. baumannii* (*ade*B). It was noted that the combination of QR + MER significantly reduced the activity of carbapenemases KPC, VIM, NDM, and OXA-type, which enabled an increase in the stability of the tested antibiotic. For acrB (+) strains, efflux pumps were inhibited in a statistically significant manner (*p* < 0.05) with a reduction in efflux of >40% (for QR = ½ MIC = 64 µg/mL). In silico docking analysis showed that QR interfered with the AcrB pump in a non-competitive manner with the PAβN. Both EPIs were binding to different critical regions of the efflux protein [127]. Furthermore, QR has demonstrated the ability to impair resistance to MER in CRAB and CRPA (carbapenem-resistant *P. aeruginosa*). The qRT-PCR analysis showed a decrease in the expression of both *ade*B and *bla_NDM_* genes [128]. De Carvalho et al. demonstrated a significant ligand interaction between QR and the structure of the gene repressor involved in the expression of the TtgABC efflux pump in the *P. putida* DOT-T1E strain. The polyphenol presented the ability to form a stable complex with the TtgR repressor. This action subsequently led to inhibition in the dissociation of the molecule from the binding area on the bacterial operon. As a result, the overcoming of resistance mediated via the efflux pump in *Pseudomonas* was revealed [129]. An in silico analysis was also performed on the docking capacity of QR and CYP comparators against a number of proteins produced by bacterial pathogens. Polyphenol was evaluated for its ability to interfere with, among others, the AdeJ protein forming the efflux pump in MDR *A. baumannii*. It has been shown that QR had a higher affinity (−7.4 kcal/mol) to the molecule than fluoroquinolone (−7.0 kcal/mol), which is an important premise in terms of the ability of polyphenol to disrupt efflux in MDR bacterial pathogens. Interestingly, polyphenol also showed a synergistic interaction with piperacillin against *P. aeruginosa* and *A. baumannii* [130]. Sahoo et al. reported on the beneficial antibacterial properties of three organic compounds derived from *Zingiber officinale*: QR, 6-gingerol, and epicatechin. In an in silico docking study, QR demonstrated the ability to block the efflux pump AcrB expressed in MDR *K. pneumoniae* [131]. The activity of Fuzheng Touxie Jiedu Huayu decoction (especially rich in QR) was examined against difficult-to-treat MDR *P. aeruginosa*, characterized by efflux pump MexAB-OprM overexpression. The in silico study evaluated the docking abilities of the major components of the decoction, including QR. Polyphenol demonstrated the ability to bind and form stable complexes with MexR and OprM proteins, which was associated with the inhibition of the function of the MexAB-OprM pump in *P. aeruginosa* [132]. QR has also been shown to have a strong affinity for the AcrB protein (model in silico), obtaining a similar binding strength as the inhibitor PAβN (−7.15 vs. −7.95 kJ/mol). Polyphenols from other tested substances (e.g., plumbagin, plumbagin nordihydroguaretic acid, shikonin, acridine) showed the highest effectiveness in inhibiting the protein of the pump expressed in the *E. coli* BW25113 strain. Interestingly, the compound itself did not have antibacterial activity against both the mentioned strain and the knock-out mutant Δ*acrB* (MIC > 1024 μg/mL) [133].

#### 2.2.2. Epigallocatechin-3-Gallate (EGCG) as an Efflux Pump Inhibitor

**Gram-negative bacteria**: The synergistic effect of epigallocatechin-3-gallate (EGCG) with β-lactam antibiotics was studied by Lee et al. Treating carbapenem-resistant *A. baumannii* (CRAB) with subinhibitory concentrations of polyphenol (128 μg/mL) resulted in the sensitization (MIC drop below 1.0 μg/mL) of all MDR strains tested. After observing a synergistic effect of EGCG with NMP (1-(1-naphtylmethyl)-piperazine), it was suggested that the effect of EGCG may be related to the inhibition of multidrug efflux pumps [134]. Kanagaratnam et al. observed a synergistic effect of EGCG with CPL and TET against *P. aeruginosa*. Subinhibitory concentrations of the substance (128 μg/mL) significantly increased the effectiveness of these classes of antibiotics. This effect was caused by the inhibition of the three-component efflux pump MexAB-OprM by the tested compound. This efflux pump system contains several potential binding sites for direct inhibitors. Since the simultaneous application of EGCG together with PAβN resulted in a significant amplification of MexAB-OprM inhibition, it has been suggested that the mechanism of EGCG’s inhibitory action is caused by the direct binding of EGCG to sites on MexAB-OprM other than those involved in PAβN binding [135]. Significant reports on the ability of EGCG to overcome resistance to erythromycin and CYP in *Campylobacter jejuni* and *Campylobacter coli* were derived from the Kurinčič et al. study. The described strains were characterized by the expression of genes encoding efflux pumps *cme*B, *cme*F, and *cme*R. EGCG at sub-inhibition concentrations (similarly to PAβN and NMP) led to a decrease in MICs for these antibiotics, demonstrating its EPI properties [136]. Kurinčič et al. also assessed the ability of EGCG to interfere with efflux pumps expressed in *C. jejuni* ATCC33559, ATCC33560, and NCTC11168 strains. The mentioned bacteria were resistant to macrolide antibiotics (e.g., erythromycin [ERT], clarithromycin [CLA], and azithromycin [AZT]). EGCG in combination with AZT and CLA led to a decrease in the MIC of antibiotics in the range of 4–64-fold in the strains forming the CmeABC and CmeDEF pumps. Importantly, polyphenol lowered the MIC of strains resistant to CLA or dyrithromycin in the range of 4–64-fold, which is an important premise in the era of increasing resistance to macrolides among *C. jejuni* strains [137].

**Gram-positive bacteria**: Fiedler et al. proved that the combination of tigecycline (TGC) and various EPIs, including EGCG, resulted in a significant increase in antibiotic sensitivity in *Enterococcus faecium* expressing MATE-type efflux pumps. Polyphenol at a concentration of 60 mg/L induced a decrease in MIC by 0.125–10-fold, as well as in the case of resistant strains positive for *van*A and *van*B gene expression [138]. EGCG also demonstrated synergistic properties with TET against *S. aureus* and *S. epidermidis* harboring the TetK efflux pump. While polyphenol itself was not particularly active against bacteria (MIC = 100 µg/mL), its subinhibitory concentration (½ MIC) in combination with TET resulted in a 256- and 128-fold decrease in the MIC of the antibiotic (for TetK[+] *S. epidermidis* and *S. aureus,* respectively). Importantly, the fluorescence study confirmed increased accumulation of the antibiotic inside *S. epidermidis* cells after prior incubation in a medium containing EGCG [139].

#### 2.2.3. Luteolin Activity as an Efflux Pump Inhibitor

**Gram-negative bacteria**: Luteolin (LT) and a number of other substances contained in Fuzheng Touxie Jiedu Huayu decoction have been tested for their ability to disrupt the activity of efflux pump MexAB-OprM in MDR *P. aeruginosa*. Similarly to the previously described QR, polyphenol also had a significant binding affinity for key efflux pump proteins MexR (−8.1 kcal/mol) and OprM (−7.4 kcal/mol) [132]. Ding et al. have shown that LT isolated from *Lophatherum gracile* Brongn. has antibacterial activity against MDR *E. coli* (harboring *sul*2, *sul*3, *gyr*A, *gyr*B, *oqx*A, and *par*C genes), yielding an MIC of 1 mg/mL. Importantly, in molecular analysis (qRT-PCR), pathogen exposure to polyphenol (1xMIC) led to a significant reduction in the expression of all these genes, including *oqx*A (a 75% decrease in expression). This gene encodes a component of an RND pump that determines resistance to, among others, fluoroquinolones [140].

**Gram-positive bacteria**: LT isolated from *Hydrastis canadensis* extract was evaluated for its ability to disrupt the NorA efflux pump function present in the *S. aureus* NCTC 8325-4 strain. The preparation showed higher antibacterial activity than the previously tested QR (75 μg/mL vs. >150 µg/mL). On the other hand, in the field of NorA pump inhibition, polyphenol obtained much lower effectiveness than the CCCP comparator (IC50 = 75 µg/mL vs. 4.7 µg/mL) [123].

**Others:** Lechner et al. tested the efficacy of LT in inhibiting efflux against *Mycobacterium smegmatis* mc^2^155. The natural preparation showed a higher antimicrobial activity by a row than resveratrol (MIC = 32 mg/L vs. 64 mg/L). Furthermore, the use of EtBr by these authors proved that the LT preparation at subinhibitory concentrations of 8 and 16 mg/L caused a twofold decrease in MIC for EtBr. Importantly, both LT and resveratrol showed no significant ability to interfere with EtBr efflux (in vitro analysis) [141]. Guo et al. revealed that after treatment with luteolin at ½ MIC, *msr*A-positive (containing msrA efflux pumps) of *Trueperella pyogenes* developed increased sensitivity to the macrolides acetylspiramycin, tylosin, and azithromycin, with MICs decreasing by 1- to 256-fold. Such a correlation was not present in the *msr*A-negative group. LT was proven to inhibit the *msr*A pump through several mechanisms: inhibition of *msr*A gene expression, interaction with post-transcriptional processing of the *msr*A gene, blocking energy acquisition by the MsrA efflux pump, and interfering with its ATP-binding regions and ATPase activity [142]. The effect of LT on the efflux systems of *T. pyogenes* was also confirmed by the study of Zhang et al. The researchers demonstrated that treatment of *T. pyogenes* with LT in concentrations of ¼ MIC led to increased sensitivity of the bacteria to GEN. Analysis of LT-treated strains showed reduced mRNA expression of MATE efflux pumps, responsible for the outflow of GEN, streptomycin, erythromycin, and roxithromycin, suggesting that inhibition occurred at the level of gene expression [143].

#### 2.2.4. Naringenin Activity as an Efflux Pump Inhibitor

**Gram-negative bacteria**: The multidrug efflux pump TtgABC, expressed by *Pseudomonas putida*, provides resistance to numerous antibiotics and toxic substances present in the environment [144]. It has been shown that substances of natural origin can interfere with the TrgR repressor molecule, which, after binding the ligand, inhibits the expression of DNA encoding the components of efflux pumps [145]. The Fernandez-Escamilla et al. study assessed whether ligand binding site mutations of TrgR affect the efficacy of the anti-efflux potentials of, among others, naringenin (NG) and phloretin [146]. For four mutant *P. putida* strains, both NG and phloretin have been shown to retain ligand-area binding capacity and form stable complexes with the repressor. Notably, phloretin (a polyphenol compound) showed the highest affinity for all altered areas of the protein in this respect [146]. Negm et al. evaluated the antibacterial activity of EtBr *thouarsii* leaf extract, rich in six compounds including NG, in relation to clinical strains and the ATCC13883 strain of *K. pneumoniae*. Furthermore, in the evaluation of the antimicrobial activity of isolated substances, NG achieved a promising MIC in the range 0.5–1 µg/mL. The extract itself showed the ability to potentiate the effects of EtBr in the cartwheel test. In addition, there was a significant decrease in the expression of genes encoding components of various efflux pumps, *nor*E, *acr*B, *mdf*A, and *yih*V, after exposure to the preparation [147]. Extremely important reports on the ability of NG to interfere with colistin resistance mediated via efflux come from the Xu et al. study. The authors showed that polyphenol and antibiotic alone were not effective against *A. baumannii* overexpressing AdeABC and AdeIJK pumps (COL MICs range: 8–32 µg/mL, NG > 512 µg/mL). However, their combination resulted in a synergistic interaction and resistance breakdown (COL MICs range: 0.25–1 µg/mL, NG: 16–32 µg/mL) [148]. Oyedara et al. examined the ability to disrupt the function of the efflux AcrAB-TolC pump present in *Salmonella enterica*. In silico molecular docking analysis showed that NG has a strong affinity for the AcrB protein (binding energy: −95.5 kJ/mol) [149].

**Gram-positive bacteria**: Although NG was not highly active against *S. aureus* SA-1199B (expression of the NorA efflux pump), reaching an MIC value > 256 µg/mL, it has been shown to be capable of interfering with the efflux mechanism. In an analysis using EtBr, polyphenol (concentration ¼ MIC) induced a fourfold decrease in MIC for EtBr. Additionally, NG also induced a fourfold decrease in MIC value for NOR (128 to 32 µg/mL) [150]. Polyphenol has also demonstrated the ability to break resistance mediated by efflux among *S. aureus* clinical strains (expressing genes: *nor*A-C, *mde*A, *lmr*S, *mep*A and *Abc*A) resistant to numerous fluoroquinolones (CYP, moxifloxacin [MOX], gatifloxacin [GTX], and ofloxacin [OFX]). The presence of pumps in the tested microorganisms was confirmed by analysis using EtBr. Exposure of the *S. aureus* 2493 strain to NG resulted in a decrease in the MIC for NOR (32 to 8 µg/mL) and erythromycin (>512 to 256 µg/mL). In turn, the combination of NG and NOR/MOX resulted in sensitivity to antibiotics of strains *S. aureus* N297214 and N307002 (NOR, respectively, 16 to 8 µg/mL and 512 to 16 μg/mL, and MOX 32-fold). Similarly, there was a spectacular decrease in MIC for NOR when combined with NG against the *S. aureus* 1079 strain (32-fold MIC reduction) [151]. It was also found that NG, similarly to another known inhibitor of efflux—chlorpromazine—may increase the activity of aminoglycoside antibiotics against *S. aureus* ATCC25923 and the SA10 strain. NG in subinhibitory concentrations (1/8 MIC = 128 µg/mL) induced a decrease in MIC for neomycin (from >600 µg/mL to <400 µg/mL) and amikacin (from ~200 to <70 µg/mL). The researchers suggested a possible role of the substance in interfering with the LmrS efflux pump, but the topic requires further in-depth research [152]. Johari et al. have shown that the simple NG derivative naringenin-4’-methyl ether present in the *Chromolaena odorata* extract exhibited efflux pump inhibitor properties in MDR *S. aureus* (three strains) and against *S. aureus* ATCC25923. Despite the lack of direct antibacterial activity (MIC ≥ 256 µg/mL), the preparation in the test using EtBr led to a decrease in the MIC of EtBr against MDR strains in the two–eightfold range [153].

**Others:** Biswas et al. have shown that polyphenol also has the ability to interfere with the function of the *Mycobacterium tuberculosis* Tap efflux pump (Rv1258c). In the in silico docking test, the preparation produced promising results, giving way to site-specific docking for only two comparators: baicalein and ellagic acid [154].

### 2.3. Non-Flavonoids Activity as an Efflux Pump Inhibitor

#### Stilbenoids

**Figure 5:**
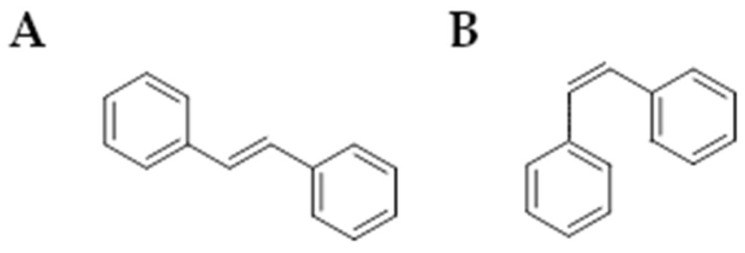
Molecular structure of stilbene isomers (**A**): *trans* (*E*)—stilbene, (**B**): *cis* (*Z*)—stilbene (two-dimensional).

Stilbenoids are a group of organic compounds of plant origin characterized by a common carbon skeleton C6-C2-C6 [155,156]. In terms of chemical structure, all substances included in this group are hydroxylated derivatives of the common precursor, stilbene (Figure 5). Other subgroups of this family are dibenzyls, bisdibenzyls, phenanthrenoids, and stilbene oligomers [157,158].

**Figure 6:**
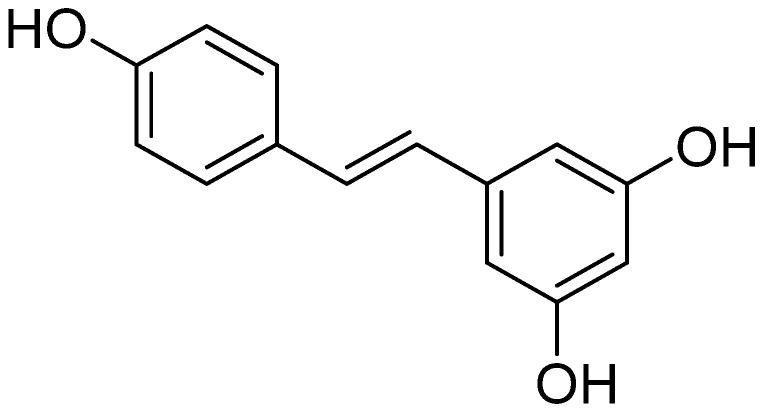
Molecular structure of resveratrol (two-dimensional).

A careful study of global literature on the EPI properties of polyphenols from the stilbenoid group has identified resveratrol (RVT) as a promising compound capable of interrupting drug resistance in bacteria. RVT (Figure 6) is a plant-derived polyphenol present in grapes, berry fruits, and nuts, as well as *Veratrum grandiflorum* and white hellebore plants [159]. It has become the subject of numerous studies on its therapeutic use, including its antiviral [160], antimicrobial [161], cardioprotective [162], neuroprotective [163], and anticancer properties [164], and its potential use in the treatment of obesity [165].

**Gram-positive bacteria**: Santos et al. have shown that RVT has the ability to interfere with the efflux pump NorA expressed by the *S. aureus* SA1199 strain and the overexpressing *nor*A gene mutant (SA1199B). RVT at subinhibitory concentrations (¼ MIC) caused a four to eightfold decrease in MIC for EtBr and induced a synergistic interaction with NOR (2–16-fold MIC reduction for fluoroquinolone). In the analysis using EtBr, after exposure to RVT of the SA1199B strain, the intensity of fluorescence caused by NorA pump inhibition was noted [166]. Another study considered the use of RVT in combating *S. aureus* resistance to NOR. It has been reported that RVT can effectively bind to the NorA efflux pump system through hydrogen bonds in some critical regions in the pump structure, leading to its inhibition [166].

**Gram-negative bacteria**: RVT isolated from *Nauclea pobeguinii* extract showed antibacterial activity against a number of Gram-negative pathogens that are resistant via efflux: *E. coli acr*F-overexpressing strains (MIC range: 32–128 µg/mL), *Enterobacter aerogenes* AcrAB-TolC harboring (MIC= 64 µg/mL), *K. pneumoniae* (MIC range: 16–64 µg/mL), *P. aeruginosa* (64–256 µg/mL), and *Providencia stuartii* AcrAB-TolC expressing (MIC range 128–256 µg/mL). Importantly, the combination of RVT at subinhibition concentrations (½ and ¼ MIC) led to the sensitization of 83.3–100% of tested strains to CYP, CPL, STM, and kanamycin and 50–66.7% to TET. A particularly significant synergistic effect was obtained with respect to the combinations of CPL + RVT and CYP + RVT (32-fold decrease of MIC) in the case of *E. coli* AG100A [167]. Abdulkareem et al. have shown that the combination of CYP and RVT can effectively reduce the expression of the *ade*F gene carried by *A. baumannii* (100% OXA-51 positive strains) derived from patients hospitalized in the ICU. RVT separately led to a twofold decrease in the expression of the gene encoding the efflux pump; in turn, its combination with CYP led to a less significant decrease to 0.93 from the primary 1.0 in the positive control (both cases had a static *p* value < 0.05) [168]. As in the 2023 study, Abdulkareem et al. evaluated the ability of RVT alone and RVT + CYP in combination to inhibit the expression of genes encoding RND-type pump components in MDR and PDR *A. baumannii* isolated from ICU ward patients. The researchers showed that exposure to RVT and RVT + CYP (at 1 × MIC concentrations) led to a decrease in *ade*I gene expression from 1.0 (unit adopted for positive control) to 0.59 and 0.79, respectively. Importantly, exposure to the antibiotic alone led to increased expression of the pump gene (to a value of 1.35). In turn, exposure to RVT alone (concentration of ¼ MIC) led to a very strong decrease in the expression of the *ade*J gene, from 1.0 (positive control) to 0.28 (~fourfold reduction) [169]. A synergistic effect of RVT with COL was observed by Cheng et al. against mcr-1-positive *E. coli* strains. The combination of RVT and COL resulted in a reduction of the starting MIC of the substance for COL in the range of 2–256-fold. As a result, in the case of five of the eight mcr-1(+) strains tested, the study showed a synergistic interaction between the compounds (FICI ≤ 0.5). The EtBr fluorescence assay revealed that RVT inhibited the activity of *E. coli* efflux pumps. However, the effect of RVT was relatively moderate compared to the combination of this compound with COL and baicalin. Molecular docking (in silico) demonstrated that the stilbenoid had an affinity for the mcr-1 protein (binding energy–5.45 kcal/mol), which confirmed the reports of the EtBr-assisted study [170]. Downregulated expression of genes encoding the AcrAB-TolC drug efflux complex in *E. coli* was also reported in an experiment by Hwang et al. The study showed that the direct target of RVT was the TolC component. RVT led to the inactivation of the *tol*C gene by inhibiting the *p-tolC* promoter [171]. Singkham et al. tested the effect of RVT against CRAB isolates. No synergistic effect was observed with RVT in combination with IMI, COL, or ACN. The combination of RVT + rifampicin was effective in 15% of *A. baumannii* cases, and RVT + chlorhexidine proved to be the most effective combination, showing synergism in all drug-resistant strains tested (FICI range of: 0.19–0.38, while FICI ≤ 0.5 was defined as synergistic). It has been revealed that the efflux pumps AdeB and AceI, responsible for the outflow of chlorhexidine in *A. baumannii*, are a key element in the synergism of these agents. It was found that significantly reduced expression of the *adeB* gene, without changes in the expression of *ace*I, *ade*R, and *ade*S, was sufficient to impair the efflux pump by RVT. Therefore, the sensitization of *A. baumannii* to chlorhexidine was primarily caused by AdeB efflux pump inhibition, while the AdeRS efflux system was not downregulated [172]. Similar findings emerged from later studies reporting that RVT inhibited the gene expression of *A. baumannii* efflux system superfamilies, AdeB and AceI. After polyphenol-based treatment, *A. baumannii* exhibited over a 10-fold decrease in the expression of *amv*A, *ade*B, and *ade*J genes and a twofold decrease in *ace*I expression [173,174].

**Others:** Lechner et al. investigated the efficacy of RVT in blocking the efflux mechanism in *M. smegmatis* mc^2^155. The preparation showed MIC at a level of 64 mg/L. Additionally, the analysis using EtBr proved that the preparation in subinhibitory concentrations of 16 mg/L caused a twofold decrease in MIC for EtBr. Resveratrol did not show a significant ability to interfere with the efflux of EtBr (in vitro analysis) [141].

### 2.4. Tannins Activity as an Efflux Pump Inhibitor

**Figure 7:**
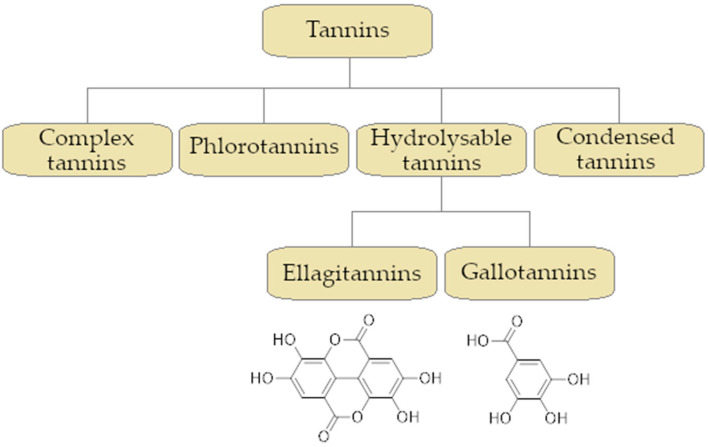
Classification of the tannins family with special regard to the hydrolysable group and the molecular structure of its monomeric precursors: ellagic acid (**left**) and gallic acid (**right**).

Tannins form an important group of polyphenolic plant-derived compounds. These substances are originally derived from various fruits, e.g., blackberries, apples, grapes, and legume trees *Acacia* spp. and *Sesbania* spp. Gallotannins are widely distributed in *Quercus infectoria, Rhus chinensis, Caesalpinia spinosa,* and *Quercus montana* [175,176]. Substances in this family are characterized by high molecular mass (500 to ≥20.000 Da) and numerous free hydroxyl phenolic groups (1–2 per 100 Da) [177,178]. Due to the presence of -OH groups, their representatives are capable of forming macromolecules or conjugates with, e.g., proteins, lipids, or saccharides [179,180]. One of the methods of tannin classification divides them into four groups: condensed tannins, hydrolysable tannins, phlorotannins, and complex tannins. This division is based on the structure of monomeric units forming a given macromolecule. Hydrolysable tannins form a polyol core (usually *D*-glucose) and esterified monomers, ellagic acid (ellagitannins) or gallic acid (gallotannins) [181,182]. Condensed tannins (proanthocyanins) are produced by polymerization of flavonoid monomers such as anthocyanins [183]. Phlorotannins are a group of tannins formed by the polymerization of phloroglucinol [184]. Complex tannins, on the other hand, are formed both as a result of the polymerization of flavonoid structures and as esters of gallic acid [185]. A summary of the tannins division into their main groups is presented in Figure 7.

#### Tannic Acid as an Efflux Pump Inhibitor

**Figure 8:**
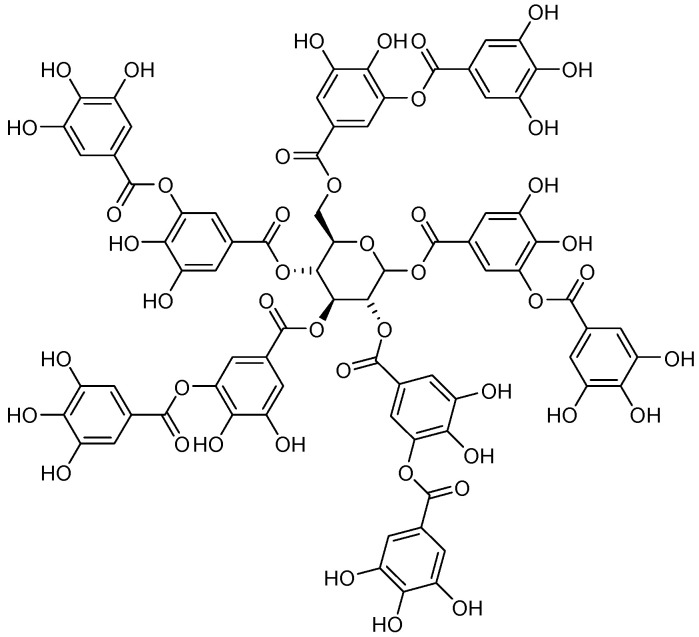
Molecular structure of tannic acid (two-dimensional).

Tannic acid (2,3-dihydroxy-5-[[(2R,3R,4S,5R,6S)-3,4,5,6-tetrakis [[3,4-dihydroxy-5-(3,4,5-trihydroxybenzoyl)oxybenzoyl]oxy]oxan-2-yl]methoxycarbonyl]phenyl] 3,4,5-trihydroxybenzoate) (Figure 8) is a representative of gallotannins, commonly extracted from the gall nuts and leaves of *Caesalpinia spinosa*, *Rhus semialata*, or *Quercus infectoria* [186]. The term tannin is ordinarily used as a synonym for tannic acid (TA). This polyphenol has numerous industrial, pharmacological, and food additive applications. Polyphenol is reported to be toxic to animals when injected into the bloodstream or consumed in large amounts, with an LD_50_ of 5 g/kg in rats and 6 g/kg in mice [187,188]. It is also a potent antioxidant [189] and has exhibited anti-mutagenic [190], antibacterial [187], and anti-inflammatory activity [191] and has reduced carcinogenic potency [188].

**Gram-positive bacteria:** Tintino et al. proved that exposure to TA induces decreased *norA* gene expression in *S. aureus* SA1199B (strain characterized by hyperexpression of the *nor*A gene). The combination of TA and NOR (both at ¼ MIC) has been shown to lead to almost complete regression of *nor*A gene expression in the SA1199B strain. Analysis with EtBr showed increased fluorescence of *staphylococcal* cells after exposure to polyphenol. Furthermore, TA has been shown to interact synergistically (FICI ≤ 0.5) with both NOR and EtBr. The in silico study indicated that TA inhibited NorA through various interactions, e.g., Van der Waals interactions, hydrogen bonds, and electrostatic effects. The ability of TA to interfere with efflux pumps can also be correlated with its effect on signaling pathways associated with the ArlRS factor (two-component regulatory system) necessary for adhesion, biofilm formation, and virulence [192]. In another study, Tintino et al. evaluated the effect of TA on *S. aureus* strains expressing two different efflux pumps: MsrA (*S. aureus* RN4220) and TetK (*S. aureus* IS-58). The preparation did not show direct antibacterial activity against both tested strains (MIC = 512 µg/mL) in contrast to, e.g., CCCP (MIC = 1–2 μg/mL) or EtBr (MIC = 32 µg/mL). The combination of TA (1/8 MIC) with antibiotics (TET, ERT) against IS-58 and RN4220 strains resulted in, respectively, a twofold reduction in MIC for TET (comparable result for TET + CCCP connection) and a greater than fourfold reduction in MIC for ERT against the MsrA harboring strain. A noteworthy combination of TA + EtBr resulted in a greater than twofold reduction in the MIC of EtBr against the IS-58 strain [187]. In a similar study, Tintino et al. evaluated the ability of polyphenol to impair the function of the NorA pump in the case of the *S. aureus* SA1199B (*nor*A gene hyperexpression) and SA1199 strains. The preparation, despite its low antibacterial activity against both strains (MIC ≥ 1024 µg/mL), limited the MIC for EtBr (>twofold) against both staph strains in a positive way. The combination of TA (1/8 MIC) and NOR resulted in a fourfold decrease in the MIC for the antibiotic (128 to 32 µg/mL) for the SA1199 strain and a spectacular MIC reduction for the SA1199B strain (~eightfold decrease) [193]. Myint et al. investigated whether TA exposure could inhibit the efflux of fusidic acid by *S. aureus* MRSA, previously exposed to subinhibitory antibiotic concentrations. Importantly, it has been shown that while chronic exposure to fusidic acid induces the development of efflux-mediated resistance in MRSA, the polyphenol (concentrations of 0.3 and 0.6 MIC) intensified EtBr accumulation inside bacterial cells. The described process can potentially protect against the development of antibiotic resistance in *Staphylococcus* [194]. Further reports on the blocking of efflux-mediated resistance in *S. aureus* via the NorA pump come from the Diniz-Silva et al. study. The researchers showed that TA (subinhibitory concentration) interfered with efflux in the SA1199B staph strain and led to an increase in NOR activity (decrease in MIC from 128 to 4 µg/mL), and the interaction was confirmed to be synergistic (FICI ≤ 0.5) [195].

**Gram-negative bacteria:** TA has been studied as a potential inhibitor of efflux pumps in MDR *A. baumannii* JVC1053 (resistant to LEV, aztreonam, CYP, and TRM-STX) and the ATCC19606 strain. The Kirby–Bauer test showed that the addition of TA in subinhibitory concentrations (40 µM) to a range of antibiotics resulted in an increase in the size of the ZOI in the case of the MDR strain for fusidic acid, rifampicin, TET, coumermycin, novobiocin, and chlorbiocin. It has also been shown that the addition of TA (40 µM) reduces the MIC for novobiocin, chlorbiocin, coumermycin (foufold), fusidic acid, and rifampicin (twofold) preparations. There was no increased accumulation of EtBr using the compound, but the increasing intracellular accumulation of pyronine Y (in vitro) proved that TA can interfere with the efflux pump system in *A. baumannii* [196]. Lin et al. conducted a study on the potential role of TA in interfering with the efflux pump system in *A. baumannii* with the active two-component BaeSR system and in the case of ΔbaeR mutants. In the case of strains lacking this gene (as in the case with other tested compounds), the inhibition of *Acinetobacter* growth was noted after TA exposure (total inhibition at 150 μg/mL). In the context of the described strain, there was a significant decrease in the expression of genes encoding efflux pump subunits *ade*B*, ade*J, and *mac*B. However, in the case of strains with functional BaeSR regulation, an increase in pump expression after TA exposure was detected (increase from a concentration of 100 µg/mL). BaeR has been identified as a potential defense mechanism that, as in the case of TGC resistance induction, can enable *A. baumannii* to survive in a TA-rich environment [197].

### 2.5. Phenolic Acids

**Figure 9:**
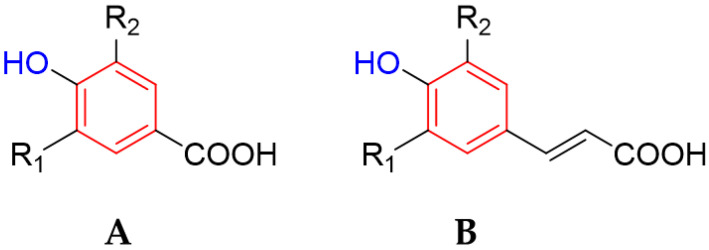
Molecular structure of hydroxybenzoic acids (**A**) and hydroxycinnamic acids (**B**).

Phenolic acids are a large group of organic compounds widely distributed in various fruits and vegetables, such as pomegranate (*Punica granatum*), raspberry (*Rubus idaeus*), carrots (*Daucus carota*), tomato (*Solanum lycopersicum*); cereals, such as wheat (*Triticum aestivum*), and rice (*Oryza sativa*); and, among others, nuts (*Juglans regia*) and coffee (*Coffea arabica*) [198,199]. From the point of view of their chemical structure, they are characterized by the presence of both carboxyl and phenolic groups [199]. In general, the group is divided into benzoic acid derivatives (hydroxybenzoic acids) and cinnamic acid derivatives (hydroxycinnamic acids) [200]. A comparison of the structure of both groups is presented in Figure 9.

#### Ellagic Acid as an Efflux Pump

**Figure 10:**
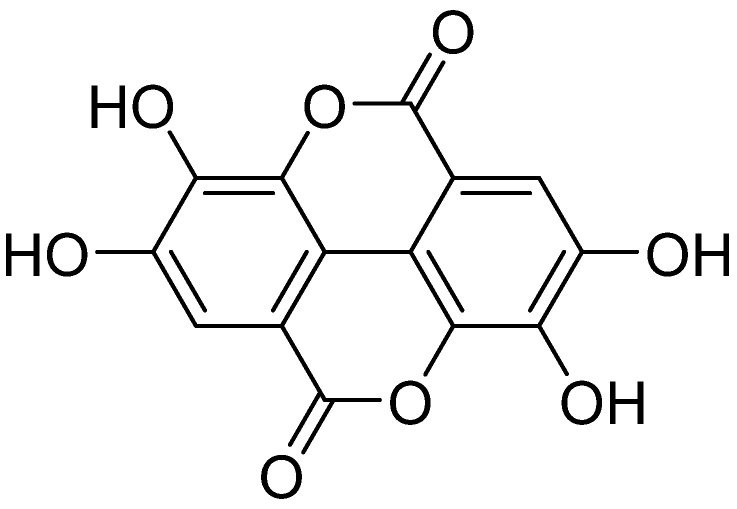
Molecular structure of ellagic acid (two-dimensional).

Ellagic acid (EA, Figure 10) belongs to the hydroxybenzoic acids and contains two oxidized and esterified molecules of gallic acid. The compound naturally occurs in many herbs and is also abundant in pomegranate (*Punica granatum* L.), grape (*Vitis vinifera* L.), and raspberry (*Rubus* spp. raspberry) [201]. EA is widely known for its antioxidant [202], antimicrobial [203], antiviral, and anti-inflammatory [204] properties. It also causes tumor gene suppression, antiproliferation, and positive regulation of apoptosis genes in cancer cells [205]. EA is insoluble in water, so its metabolites are characterized by low bioavailability and are quickly eliminated. Therefore, its therapeutic advantage is limited, since most clinical drugs are administered orally [206].

**Gram-negative bacteria**: EA was identified as a potential EPI active against MDR *A. baumannii* JVC1053 (resistant to LEV, aztreonam, CYP, and TRM-STX) and ATCC19606 strains. The exposure to polyphenol (40 µM) led to an increase in the ZOI diameter of antibiotics (novobiocin, chlorbiocin, coumermycin, TET, RIF, and fusidic acid) tested against the MDR strain (Kirby–Bauer assay). EA (40 µM) was revealed to reduce the MIC for novobiocin, coumermycin, fusidic acid, and rifampicin (fourfold) preparations. Moreover, the 100% reduction in the growth of the MDR *A. baumannii* strain was noted with the combination of EA (40 µM) and novobiocin (1/4 MIC). This activity (both substances at subinhibitory concentrations) suggested that polyphenol may interrupt efflux-mediated resistance among *Acinetobacter*. There was no increased accumulation of EtBr using the compound, but the increasing intracellular accumulation of pyronine Y (in vitro) proved that EA disrupted the efflux pump system in *A. baumannii* [196].

**Gram-positive bacteria**: Macêdo et al. performed a test using EA and gallic acid, which showed a reduction in the MIC value of TET from 256 to 128 μg/mL for the IS-58 *S. aureus* strain. Additionally, in the test with CYP in the presence of EA and gallic acid, antibacterial activity was increased—an MIC reduction from 161.27 to 128 μg/mL. It has been proven that neither acid showed direct activity on the efflux pump in *S. aureus* but their properties were associated with affecting *tet*K and *mep*A gene expression. However, the authors did not explain the specific mechanism of action, so further molecular tests are needed [207].

**Others:** Due to *M. tuberculosis* acquiring resistance to RIF, Nirmal et al. conducted a study of EA that showed synergistic inhibitory activity with RIF against resistant *Mycobacterium* strains. In the in silico assay, the possibility of inhibiting the Rv1819c- BecA efflux pump was noted. EPI activity was performed by evaluating the accumulation of EtBr in MDR strains of *M. tuberculosis* and the BecA-expressing recombinant *M. smegmatis* strain. The cytotoxicity of the tested inhibitors (including EA) was assessed using the in silico and ex vivo methods. Recombinant *M. smegmatis* exposed to EtBr in the presence of EA (0.5 MIC concentration) showed increased accumulation of EtBr. EA exposure led (among other EPIs like piperine) to the strongest BecA inhibition (fluorescence intensity assay). This ability of EA to interfere with BecA EP presents a strong premise for its EPI properties in disrupting RIF resistance [208]. An in silico study performed by Biswas et al. showed the potential of EA to bind to the efflux pump (Rv1258c) Tap, which is responsible for the resistance of *M. tuberculosis* to various tuberculostatics. EA exhibited significant binding affinity to the structure of Tap EP via the formation of, e.g., hydrogen bonds. The polyphenolic compound showed satisfactory docking results and bioavailability as a possible EPI active against MDR *M. tuberculosis* [154].

## 3. Cutting-Edge Technologies Enhancing the Efficiency of Plant-Derived Drugs

The newly synthesized derivatives of the phytochemicals described above offer great hope in the fight against growing bacterial resistance to antibiotics associated with active pumping out. The EPI properties of these polyphenolic derivatives demonstrated in vitro make it possible to successfully use them in modern nanotechnologies. Table 3 is dedicated to summarizing information on the anti-efflux properties of polyphenolic derivatives.

One of the most rapidly developing nanotechnologies in phytomedicine in recent times is the process of synthesizing nanoparticles (NPs). Due to the chemical characteristics of substances of natural origin it is possible to use them in both the synthesis of metal-based NPs (green synthesis, based on their reducing and stabilizing properties) and the formation of compound-based NPs [215,216]. Table 4 summarizes data on the nanotechnology implications of the compounds described in this review.

## 4. Materials and Methods

In this review, we searched for articles in the Scopus, PubMed, Web of Science, and Google Scholar databases. In total, 225 articles were cited. The articles were qualified for review by searching for the following keywords in their titles and abstracts: “efflux pump”, “curcumin”, “quercetin”, “luteolin”, “naringenin”, “tannic acid”, “ellagic acid”, “resveratrol”, “epigallocatechin-3-gallate”, “stilbenoids’’, “flavonoids’’.

Our review includes articles that were published after 2012. Older manuscripts were classified as not qualifying and were rejected.

Figure 1 presents a simplified scheme of the molecular action of several classes of efflux pumps. Figure 2 presents the classification of natural polyphenolic compounds based on structure. Figure 3, Figure 5, Figure 6, Figure 8 and Figure 10 illustrate the molecular structures of the polyphenols qualified for review. Figure 4 presents the classification of flavonoids, and Figure 7 presents the classification of tannins. Figure 9 shows the simplified molecular formula of phenolic acids.

Table 1 describes the classification, prevalence, and substrates of the efflux pumps depicted in Figure 1. Table 2 describes the characteristics of qualified flavonoids and their properties. Table 3 and Table 4 present summarized data on the EPI properties of polyphenols’ derivatives and polyphenols’ applications in nanomedicine, respectively. The symbols used represent the following: ↓—down regulation/decrease in; ↑—up regulation/increase in.

## 5. Conclusions

Several types of EPIs with documented efficacy (CCCP, PAβN, NMP) have already been recognized, but their excessive toxicity unfortunately excludes them from clinical practice. The evidence we presented about the effectiveness of plant-derived polyphenols in this field opens the door to discussing their role in this issue. The described compounds are characterized by low systemic toxicity and are widely used in traditional and herbal medicine. The fact that many of them (especially quercetin) presented inhibitory activity comparable to that of reserpine or verapamil is extremely important. On reflection, it appears that synthesizing polyphenol derivatives as a novel EPI and pursuing synergy with classic antibiotics is a promising strategy. The reports on the ability of flavonoids (QR, EGCG, and LT) to interfere with the function of the MexAB-OprM efflux pump, e.g., in non-fermenting bacilli such as *P. aeruginosa,* are especially significant. The mentioned pump is characterized by a very broad substrate spectrum, which poses a serious threat of rapidly developing multidrug resistance. QR, RVT, and EA also appear to be comparatively promising, with the ability to downregulate the expression of key genes encoding components of the AdeABC pump, present especially in MDR *A. baumannii*. Both cited pathogens are responsible for the vast majority of infections in the ICU, and are often characterized by limited treatment options, which makes these reports all the more clinically valuable. What is more, rapid molecular diagnostic systems, e.g., MALDI-TOF MS, were developed to be capable of confirming the presence of efflux pumps in detected bacteria. A successful way to overcome multidrug resistance may be achieved by combining this possibility with the simultaneous use of novel EPIs and classic antibiotics. However, to achieve this targeted therapy, more research in this field is required.

## Figures and Tables

**Table 1:** Summary of efflux pumps classification including spectrum of antibiotic resistance.

Efflux Pump Superfamily	Pumps Examples	Main Producers	Antibiotic Resistance Profile	References
RND (Resistance- Nodulation-Cell Division)	AcrAB-TolC, AdeABC, MexAB-OprM, MexCD-OprJ, MmpL5, MtrCDE, OqxAB, SmeABC	*M. tuberculosis* **GNB**: *N. gonorrhoeae*, *Enterobacteriaceae,* **N-F**: *P. aeruginosa,* *A. baumannii,* *S. maltophilia*	AMG, CPL, FLQ, LNC, MCL, PLX, RIF, SLF, TET, BL	[17,18,19,20,21,22,23]
MATE (Multidrug and toxic compound extrusion)	AbeM, DinF, EmmdR, FepA, MepA, NorM, KetM, PmpM, Rv2836c	*M. tuberculosis* **GNB**: *N. gonorrhoeae,* *K. pneumoniae* **GPB**: *S. aureus,* *L. monocytogenes*, *E. faecalis* **N-F**: *A. baumannii,* *P. aeruginosa*	AMG, CPL, FLQ, TGC, TRM	[17,24,25,26,27,28]
ABC (ATP-binding cassette)	AbcA, EfrAB, LmrA, macAB-TolC, MsrA, PatA/B, Rv1218c	*M. tuberculosis,* **GPB** *: E. faecalis,* *S. pneumoniae, S. aureus* **GNB** *: Enterobacteriaceae*	AMG, CPL, FLQ, LNC, MCL, RIF, TET	[17,29,30,31,32,33,34]
MFS (Major facilitator superfamily)	AbaQ, EmrAB-TolC, Lde, MdeA, MdfA, Mef, NorA-D, QepA, Rv1258c, TetK	*M. tuberculosis* **GPB**: *S. aureus,* *S. pneumoniae,* *L. monocytogenes*, **GNB**: *Enterobacteriaceae* **N-F**: *P. aeruginosa,* *A. baumannii*	AMG, CPL, COT, FLQ, LNC, MCL, PLX, RIF, SLF, TET, TGC, BL	[17,35,36,37,38,39]
SMR (Small multidrug resistance family)	AbeS, EmrE, KpnEF, Mmr, QacC-J, SepA	*M. tuberculosis* **GPB**: *S. aureus,* *E. faecalis* **GNB**: *Enterobacteriaceae* **N-F**: *P. aeruginosa,* *A. baumannii*	AMG, MCL, PLX, RIF, SLF, TET, BL	[8,17,40,41,42]

Abbreviations: AMG—aminoglycosides, BL—β-lactams, CPL—chloramphenicol, COT—cotrimoxazole, FLQ—fluoroquinolones, GNB—Gram-negative bacteria, GPB—Gram-positive bacteria, LNC—lincosamides, MCL—macrolides, N-F—non-fermenting rods, PLX—polymyxins, RIF—rifampicin, SLF—sulfonamides, TET—tetracyclines, TGC—tigecycline, TRM—trimethoprim. Strains: *A. baumannii*—*Acinetobacter baumannii*, *E. faecalis*—*Enterococcus faecalis*, *K. pneumoniae*—*Klebsiella pneumoniae*, *L. monocytogenes*—*Listeria monocytogenes*, *M. tuberculosis*—*Mycobacterium tuberculosis*, *N. gonorrhoeae*—*Neisseria gonorrhoeae*, *P. aeruginosa*—*Pseudomonas aeruginosa*, *S. aureus*—*Staphylococcus aureus*, *S. maltophilia*—*Stenotrophomonas maltophilia*, *S. pneumoniae*—*Streptococcus pneumoniae*.

**Table 2:** Characteristics of flavonoids characterized by EPI properties.

Structure and Name	Flavonoid Group	Plant Cultivar	Biological Activity	References
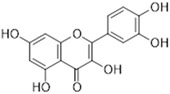 Quercetin	Flavonol	*Moraceae*, *Theaceae*, *Apiaceae*, *Brassicaceae*, *Asteraceae, Rosaceae*	anti-inflammatory anti-microbial anti-neoplasm gastroprotective	[95,96,97,98,99]
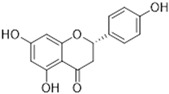 Naringenin	Flavanone	Main source: *Citrus aurantium,* *Citrus clementia,* *Citrus reticulata*	antidiabetic anti-estrogenic anti-inflammatory anti-neoplasm antioxidant hypolipidemic neuroprotective	[100,101,102,103,104]
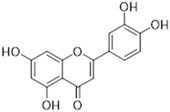 Luteolin	Flavone	Various fruits and vegetables e.g., *Apium graveolens* L., *Chrysanthemum* L. and *Daucus carota* Hoffm.	antidiabetic anti-inflammatory anti-microbial anti-neoplasm antioxidant cardioprotective	[105,106,107,108,109,110]
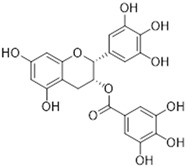 Epigallocatechin-3-gallate	Flavan-3-ol	Main source: *Camellia sinensis* (L.) Kuntze	anti-inflammatory anti-microbial anti-neoplasm antioxidant anti-viral neuroprotective	[111,112,113,114,115,116,117,118,119,120,121,122]

**Table 3:** Summary of polyphenol-based derivatives’ anti-efflux activity.

Name of Derivative	Precursor Polyphenol	Pathogens	Mechanism of Antimicrobial Activity	References
1,3-Bis-(E/E)-[3-(3-Methoxy,4-hydroxyphenyl)- 1-oxo-2-en-yl]- benzene	CU	*P. aeruginosa* (MBL positive)	Synergy with TET	[209]
			↓ expression of *mex*A, *mex*B and *opr*M genes (separately and combined with TET)	
			Direct inhibition of pumps subunits: MexA, MexB, OprM	
(1E,4E)-1,5-Di (thiophen-2-yl) penta-1,4-dien-3-one		*S. aureus* 1199B and K2068	Synergy with NOR	[210]
			Direct inhibition of pumps: NorA (1199B) and MepA (K2068)	
quercetin 7-*O*-glutamate	QR	*S. aureus*	Inhibition of NorA pump (superior to QR and comparable to reserpine)	[211]
			Potentiating of antibiotics activity for: TET (2-fold MIC reduction), CYP and GEN (4-fold)	
3-*O*-substituted quercetin		CRE	Direct inhibiton of AcrB pump subunit	[212]
			Inhibition of MBL e.g., NDM-1	
			Synergy with MER (>4-fold MIC reduction)	
Naringenin- ethylidene- fluoroquinolone hybrid	NG	*E. coli* *Bacillus subtilis* *S. aureus*	Synergy with CYP (8–88-fold MIC reduction)	[213]
			Enhancing bacterial DNA-gyrase inhibition	
			Reduction in CYP efflux ratio in tested strains	
Naringin dihydrochalcone	NG	CRAB	Direct inhibition of AdeB protein	[214]
			Inhibition of MER efflux	

Abbreviations: CRAB—carbapenem-resistant *A. baumannii*, CRE—carbapenem-resistant *Enterobacteriaceae*, CU—curcumin, CYP—ciprofloxacin, GEN—gentamycin, MBL—metallo-β-lactamase, MIC—minimal inhibitory concentration, MER—meropenem, NDM-1—New Delhi MBL type 1, NG—naringenin, NOR—norfloxacin, QR—quercetin, TET—tetracycline.

**Table 4:** The summary of selected polyphenols’ implications for nanomedicine.

Polyphenol Precursor	Nanotechnology Application	Pathogens	Mechanism of Antibacterial Action	References
CU	Polyphenol-based NPs	MDR *P. aeruginosa* and PA01 strain	↓ expression of *mex*B, *mex*T, *mex*D genes	[217]
			↑ expression of *nfx*B gene (negative regulator of MexCD-OprJ)	
		CYP-NS *P. aeruginosa*	Concentration-dependent enhancing activity of CYP by NPs	[218]
			Increased accumulation of CYP in pathogen cells	
			↓ expression of *mex*X and *opr*M genes (mexXY-oprM and mexAB-oprM pumps)	
	CU-based NPs combined with 2,3-dimethyl maleic anhydride	MDR *P. aeruginosa*	↓ expression of *mex*A-F and *opr*M genes	[219]
			Synergy with TOB	
	Polyphenol-based NPs	*P. aeruginosa*	Synergy with GEN (combination with CU formed ZOI comparable to combination with verapamil)	[220]
	CU-stabilized silver NPs (green synthesis)	*S. aureus* MRSA	↓ expression of *sdr*M gene	[221]
	CU-synthesized samar oxide-based NPs (green synthesis)	MDR *S. aureus*	↓ expression of *nor*A-B genes	[222]
		MDR *P. aeruginosa*	↓ expression of *mex*A-B genes	
NG	naringin dihydrochalcone based silver NPs	CRAB	Generating of ROS and RNS	[214]
			Increasing susceptibility to MER via disrupting transmembrane proton gradient (malfunction of RND type pumps e.g., AdeABC)	
QR	QR-stabilized silver NPs (green synthesis)	*S. aureus*	Enhanced EtBr accumulation	[223]
			↓ expression of *nor*A-C genes	
TA	TA-stabilized silver NPs (green synthesis)	*Burkoholderia pseudomallei* MDR *Enterobacter cloacae*	↑ permeability of cell membrane via silver NPs	[224]
			Interference with acrAB-TolC efflux pump	
	TA-based hybrid NPs	*S. aureus* MRSA, *P. aeruginosa*	NorA pump inhibition via metal ions chelating	[225]
			Inhibition of *QS*	
EA	Polyphenol-bonded magnetic Fe_3_O_4_ NPs	ESβL *E. coli*	Eradication both planktonic and biofilm form of pathogen	[206]
			↓ expression of *acr*B and *tol*C genes	

Abbreviations: CRAB—carbapenem-resistant *A. baumannii*, CU—curcumin, CYP—ciprofloxacin, EA—ellagic acid, ESβL—extended-spectrum β-lactamase, EtBr—etidine bromide, GEN—gentamycin, MDR—multidrug-resistant, MER—meropenem, MRSA—methicillin-resistant *S. aureus*, NG—naringenin, NPs—nanoparticles, NS—non-susceptible, *QS*—*quorum sensing*, QR—quercetin, RND—resistance-nodulation cell division efflux pump type, RNS—reactive nitrogen species, ROS—reactive oxygen species, TA—tannic acid, TOB—tobramycin, ZOI—zone of inhibition.
